# In vivo characterization of *candida* extracellular vesicles reveals unique infection pathway proteins

**DOI:** 10.1172/jci.insight.198953

**Published:** 2026-04-23

**Authors:** Justin Massey, Robert Zarnowski, William Hartman, Jeniel Nett, David Andes

**Affiliations:** University of Wisconsin, Madison, Wisconsin, USA.

**Keywords:** Infectious disease, Microbiology, Fungal infections

## Abstract

The study describes the in vivo isolation, characterization, and relevance of fungal biofilm extracellular vesicles.

Extracellular vesicles (EVs) are membrane-bound particles secreted from cells across all kingdoms of life (1, 2). They are implicated in trafficking of molecules between cells and, therefore, affect function of surrounding cells. Microbial EVs can affect both microbial and host cells, modulating infection outcome. Since these EVs carry complex cargo, it is often challenging to identify specific EV component(s) responsible for individual functional effects. Furthermore, the similarities in EV size and surface properties between microbial and host EVs preclude use of general isolation techniques for cell specific separation (3). An additional obstacle to isolation and study of microbial derived EVs is the relatively low concentrations of microbe EVs compared with host EVs in the infection environment. The ability to capture and characterize EVs in vivo has proven important for study of disease mechanisms and has been utilized for diagnostic purposes in cancer biology (1). However, successful application of in vivo EV investigation to other areas of study, including infectious diseases, has been limited.

One distinction between microbial and host-produced EVs is their proteome contents, which include proteins that decorate the EV surface (4). These surface protein differences can be exploited for immunoaffinity capturing (IAC) isolation of cell specific EVs. We have previously shown EVs from *Candida* biofilms contain distinct protein components, including those adorning the EV surface (5). In the present investigation we isolated *C*. *albicans* EVs from in vitro propagated biofilms and generated polyclonal antibodies to the EVs in rabbits (Rb-CA-EV-pAbs) (Figure 1A). Subsequent Western blotting and super-resolution microscopy of in vitro–isolated *Candida* EVs demonstrated the successful capture of the fungal EVs in a concentration-dependent manner (Figure 1, B and C). Sequencing of the 12 kDa band revealed numerous putative EV surface antigen targets (Supplemental Table 1; supplemental material available online with this article; https://doi.org/10.1172/jci.insight.198953DS1), including proteins previously identified in biofilm EV analysis (4, 5). We then explored the utility of the antibodies to isolate and characterize *Candida* EVs from an in vivo rat catheter model that mimics a common device biofilm in patients. Using a low volume (10 μL) of blood from the rat catheter biofilm, we find the IAC methodology captures *Candida* EVs (Figure 1D). Further assessment of lipid components identified unique fungal cerebroside biomarkers (Figure 1E). We also find the Rb-CA-EV-pAbs bind to biofilm EVs from other clinically common *Candida* species (Supplemental Figure 1A) (5) and interact with EVs from multiple *C*. *albicans* clinical isolates (Supplemental Figure 1B). We then assessed the ability to release intact EVs for characterization of their size, protein composition and quantity. Nanoparticle tracking analysis of in vivo–isolated EV from uninfected controls and *Candida* biofilm demonstrate a relatively large quantity of EVs from both conditions prior to IAC (Figure 1F). However, EV quantity from the *Candida* biofilm catheter samples demonstrated an enrichment of EVs following IAC. The size of the EVs was approximately 100 nm, consistent with an exosome size; this is the predominant population during biofilm conditions in vitro (Figure 1, G and H). Uninfected rat plasma controls did not identify EVs, providing further evidence of specificity. We next explored the in vivo function of a *Candida* mutant that was previously found to be important for vesicle production in vitro (Hse1ΔΔ) (4). We used imaging flow cytometry to quantify the EVs and found the mutant strain EVs were over 5-fold reduced compared with the parent strain during rat biofilm infection (Figure 1, I and J, and Supplemental Figure 1C). The congruent findings demonstrate the utility of the methodology for in vivo validation of in vitro observations. We further examined the EV proteome from the purified in vivo *Candida* EV sample (Figure 1K and Supplemental Tables 1 and 2, Supplemental Methods). Among the *Candida* proteins identified, we note a subset that had previously been identified in EVs from in vitro biofilms and the biofilm matrix, validating the model (5). For example, we found DOA4, an ESCRT pathway component and EV marker, previously shown to be important for biofilm matrix production and function. Strikingly, our in vivo EV analysis revealed numerous proteins unique to the in vivo environment, suggesting potentially novel EV cargo loading in response to the host environment. Among the 34 unique cargo, we identified a kinase (Cbk1) with recently demonstrated function in this in vivo catheter biofilm model (6). We speculate that this may be serving as a sensor of an in vivo environmental condition. In sum, we find that IA-targeting *Candida* biofilm EVs allows for specific capture of fungal EVs from an infected mammalian host. The EVs can be released for analysis of EV quantity, composition, and function. Application to other microbes may complement available diagnostic and pathogenesis investigation in common infectious diseases.

## Conflict of interest

The authors have declared that no conflict of interest exists.

## Funding support

This work is the result of NIH funding, in whole or in part, and is subject to the NIH Public Access Policy. Through acceptance of this federal funding, the NIH has been given a right to make the work publicly available in PubMed Central.

- NIH NIAID R01 AI073289-16 to David Andes.

## Supplementary Material

Supplemental data

Unedited blot and gel images

Supporting data values

## Figures and Tables

**Figure 1:**
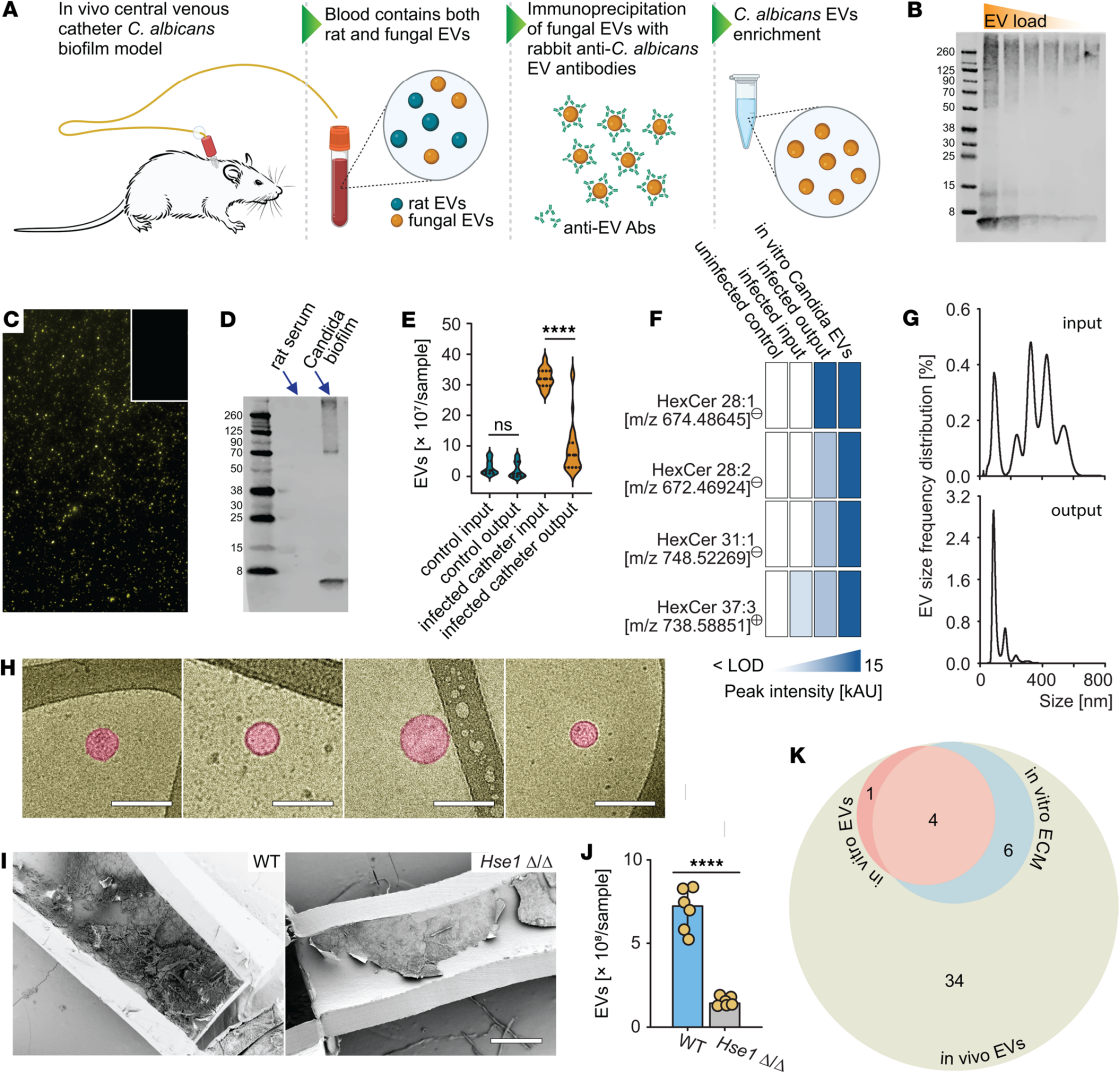
Isolation and characterization of *Candida* EVs from an in vivo vascular catheter biofilm. (**A**) A schematic workflow of the EV isolation protocol from i.v. catheters of rats infected with *Candida*. Following blood collection, fungal EVs were isolated and enriched via IA using Rb-CA-EV-pAbs. (**B**) Western blot titration of *C*. *albicans* biofilm EVs by Rb-CA-EV-pAbs. (**C**) IAC of *C*. *albicans* EVs with Rb-CA-EV-pAbs visualized by super-resolution microscopy. Inset: no EV signal was detected in the absence of Rb-CA-EV-pAbs. Scale bar: 1.5 µm. (**D**) Western blot analysis of in vivo *C*. *albicans* EVs in uninfected control and *C*. *albicans*–infected rat serum EVs by Rb-CA-EV-pAbs. No EV signal was detected in uninfected rat sera. (**E**) NTA-based concentrations of EVs in unprocessed (input) and processed (output) rat serum samples collected from intravenous catheters (*n* = 3, *P* < 0.001). (**F**) Mass spectrometry–based analysis of fungal-unique biomarker cerebrosides. (**G**) NTA-based sizing of EVs in unprocessed (input) and processed (output) rat blood samples from *Candida* biofilm i.v. catheters. (**H**) Representative cryoEM images showing in vivo EVs isolated from *Candida*-infected rat catheter. Scale bar: 100 nm. (**I**) Scanning electron micrographs of *C*. *albicans* biofilms of WT and ESCRT-associated *hse1ΔΔ* mutant strains from rat catheters. Scale bar: 400 μm. (**J**) Imaging flow cytometry quantitative analysis of in vivo EVs in the *C*. *albicans* of WT and ESCRT-associated *Hse1ΔΔ* strains from rat venous catheters (*n* = 4, 6 technical each). Kruskal-Wallis 1-way ANOVA, followed by uncorrected Dunn’s multiple-comparison test. ****P < 0.001. (**K**) Venn diagram depicting the qualitative profiling of in vitro and in vivo *C*. *albicans* proteomes.

## Version 1 04/23/2026

In-Press Preview

## Version 2 06/08/2026

Electronic publication
