# Supplementary material for: In vivo characterization of candida extracellular vesicles reveals unique infection pathway proteins
Source: JCI Insight. 2026 Apr 23;11(11):e198953. doi: 10.1172/jci.insight.198953 (PMC13317910; doi:10.1172/jci.insight.198953)
Supplement: Supplemental data [file jciinsight-11-198953-s002.pdf]

**Supplemental Information**

# In Vivo Characterization of *Candida* Extracellular Vesicles Reveals Unique Infection Pathway Proteins and Validates In Vitro Observations

Justin Massey, Robert Zarnowski, William Hartman, Jeniel Nett, David Andes

**Table S1.** ***C. albicans*** **EV proteins identified in the 12 kDa PAGE/WB band.**

| UniProt ID# | Gene name | Gene ORF# | Description |
|---|---|---|---|
| A0A1D8PHC9 | AAT1 | orf19.3554 | Aspartate aminotransferase; soluble protein in hyphae; macrophage-induced protein; alkaline upregulated; amphotericin B repressed; gene used for strain identification by multilocus sequence typing; farnesol-, Hap43p-induced; GlcNAc-induced |
| Q59XW4 | ACS1 | orf19.1743 | Acetyl-CoA synthetase; induced by human neutrophils; fluconazole-repressed; regulated by Nrg1/Mig1; colony morphology-related gene regulation by Ssn6; only in stationary phase cultures; rat catheter biofilm repressed, Spider biofilm induced |
| A0A1D8PFR4 | ACT1 | orf19.5007 | Actin; gene has intron; transcript regulated by growth phase, starvation; at polarized growth site in budding and hyphal cells; required for wild-type Cdc42 localization; unprocessed N terminus; Hap43-induced; Spider biofilm repressed |
| A0A1D8PP43 | ADH1 | orf19.3997 | Alcohol dehydrogenase; oxidizes ethanol to acetaldehyde; at yeast cell surface; appears to have a regulatory role in arginine catabolism and hyphal growth; immunogenic in humans/mice; fluconazole, farnesol-induced; flow model biofilm induced |
| O94038 | ADH2 | orf19.5113 | Alcohol dehydrogenase; soluble in hyphae; expression regulated by white-opaque switching; regulated by Ssn6; indued by Mnl1 in weak acid stress; protein enriched in stationary phase yeast cultures; Spider biofilm induced |
| A0A1D8PGT5 | ALD5 | orf19.5806 | NAD-aldehyde dehydrogenase; decreased expression in fluconazole-resistant isolate, or in hyphae; biofilm induced; fluconazole-downregulated; protein abundance is affected by URA3 expression in the CAI-4 strain; stationary phase enriched |
| O94083 | ANB1 | orf19.3426 | Translation initiation factor eIF-5A; repressed in hyphae vs yeast cells; downregulated upon phagocytosis by murine macrophage; Hap43-induced; GlcNAc-induced protein; Spider biofilm repressed |
| A0A1D8PG50 | ATP18 | orf19.2066.1 | F1F0 ATP synthase complex subunit; fungal-specific; gene has intron |
| A0A1D8PKZ9 | ATP2 | orf19.5653 | F1 beta subunit of F1F0 ATPase complex; antigenic in human, mice; induced by ciclopirox olamine; caspofungin repressed; macrophage/pseudohyphal-induced; detected during exponential and stationary growth phases; Spider biofilm repressed |
| Q59PV8 | ATP7 | orf19.2785 | Putative subunit of the F1F0-ATPase complex; colony morphology-related gene regulation by Ssn6; farnesol, macrophage-downregulated protein abundance; protein present in exponential and stationary yeast growth phases; Hap43-induced |
| O42766 | BMH1 | orf19.3014 | Sole 14-3-3 protein in C. albicans; role in hyphal growth; possibly regulated by host interaction; localizes to yeast-form cell surface, not hyphae; alternatively spliced 5' UTR intron; Spider biofilm repressed |
| A0A1D8PKC3 | CAM1 | orf19.7382 | Putative translation elongation factor eEF1 gamma; protein level decreased in stationary phase cultures; Spider biofilm repressed |
| A0A1D8PL14 | CAR2 | orf19.5641 | Ornithine aminotransferase; arginine metabolism; alkaline induced; mutant sensitivite to toxic ergosterol analog, to amphotericin B; exponential and stationary phase yeast; flow model biofilm induced; rat catheter, Spider biofilm repressed |
| O13289 | CAT1 | orf19.6229 | Catalase; resistance to oxidative stress, neutrophils, peroxide; role in virulence; regulated by iron, ciclopirox, fluconazole, carbon source, pH, Rim101, Ssn6, Hog1, Hap43, Sfu1, Sef1, farnesol, core stress response; Spider biofilm induced |
| P46614 | CDC19 | orf19.3575 | Pyruvate kinase at yeast cell surface; Gcn4/Hog1/GlcNAc regulated; Hap43/polystyrene adherence induced; repressed by phagocytosis/farnesol; hyphal growth role; stationary phase enriched; flow model biofilm induced; Spider biofilm repressed |
| P40953 | CHT2 | orf19.3895 | GPI-linked chitinase; required for normal filamentous growth; repressed in core caspofungin response; fluconazole, Cyr1, Efg1, pH-regulated; mRNA binds She3 and is localized to yeast-form buds and hyphal tips; Spider biofilm repressed |
| A0A1D8PSH3 | CIT1 | orf19.4393 | Citrate synthase; induced by phagocytosis; induced in high iron; Hog1-repressed; Efg1-regulated under yeast, not hyphal growth conditions; present in exponential and stationary phase; Spider biofilm repressed; rat catheter biofilm induced |
| A0A1D8PP59 | COR1 | orf19.4016 | Putative ubiquinol-cytochrome-c reductase; amphotericin B induced; repressed by nitric oxide, Hap43p, and Spider biofilm; null mutant is viable but shows decreased vegetative growth on several carbon sources |

| | | | |
|---|---|---|---|
| Q5APK5 | COX5 | orf19.4759 | Cytochrome oxidase subunit V; putative upstream CCAAT box regulatory element; macrophage/pseudohyphal-induced; repressed by nitric oxide; intron in 5'-UTR; Hap43p-dependent repression in low iron medium |
| P53698 | CYC1 | orf19.1770 | Cytochrome c; complements defects of S. cerevisiae cyc1 cyc7 double mutant; induced in high iron; alkaline repressed; repressed by nitric oxide; Hap43-dependent repression in low iron; regulated by Sef1, Sfu1 |
| P22011 | CYP1 | orf19.6472 | Peptidyl-prolyl cis-trans isomerase; cyclosporin A sensitive activity; soluble in hyphae; biofilm induced, macrophage-induced protein; downregulated upon treatment of biofilm with farnesol; present in exponential and stationary phase cells |
| Q5AAW3 | DHH1 | orf19.6197 | Putative RNA helicase |
| A0A1D8PHE5 | ECS2 | orf19.6867 | Protein involved in resistance to caspofungin and anidulafungin; has a predicted cytochrome b5-like heme/steroid binding domain; repressed by alpha pheromone in SpiderM medium |
| Q5A0M4 | EFT2 | orf19.5788 | Elongation Factor 2 (eEF2); GTPase; essential; highly expressed; target of sordarin antifungals; antigenic in human/mouse; lacks site for regulatory phosphorylation by eEF2 kinase; GCN-regulated; higher protein amount in stationary phase |
| Q5ANP2 | EGD2 | orf19.5858 | Nascent polypeptide associated complex protein alpha subunit; soluble protein in hyphae; macrophage/pseudohyphal-induced; protein level decrease in stationary phase cultures; GlcNAc-induced protein; Spider biofilm repressed |
| P30575 | ENO1 | orf19.395 | Enolase, involved in glycolysis and gluconeogenesis; also has transglutaminase activity involved in assembly of cell wall polysaccharides; major cell-surface antigen; binds host plasmin/plasminogen; immunoprotective; may be essential |
| A0A1D8PH78 | ERG20 | orf19.4491 | Putative farnesyl pyrophosphate synthetase involved in isoprenoid and sterol biosynthesis, based on similarity to S. cerevisiae Erg20p; likely to be essential for growth, based on an insertional mutagenesis strategy |
| Q9URB4 | FBA1 | orf19.4618 | Fructose-bisphosphate aldolase; glycolytic enzyme; antigenic in murine/human infection; regulated by yeast-hypha switch; induced by Efg1, Gcn4, Hog1, fluconazole; phagocytosis-repressed; flow model biofilm induced; Spider biofilm repressed |
| A0A1D8PKW2 | FBP1 | orf19.6178 | Fructose-1,6-bisphosphatase; key gluconeogenesis enzyme; regulated by Efg1, Ssn6; induced by phagocytosis; effects switch from glycolysis to gluconeogenesis in macrophage; rat flow model biofilm induced; overlaps orf19.6179 |
| Q59QN6 | FDH1 | orf19.8252 | Formate dehydrogenase; oxidizes formate to CO2; Mig1 regulated; induced by macrophages; fluconazole-repressed; repressed by Efg1 in yeast, not hyphal conditions; stationary phase enriched; rat catheter and Spider biofilm induced |
| A0A1D8PKV4 | FUM12 | orf19.6724 | Putative fumarate hydratase; enzyme of citric acid cycle; fluconazole, Efg1 repressed; induced in high iron; protein present in exponential and stationary growth phase |
| A0A1D8PI00 | GDH2 | orf19.9738 | Mitochondrial NAD-dependent glutamate dehydrogenase; catalyzes deamination of glutamate to alpha-ketoglutarate; fungal-specific; regulated by Nrg1p, Mig1p, Tup1p, and Gcn4p; stationary phase enriched; Spider and flow model biofilm induced |
| Q59TZ8 | GLK1 | orf19.734 | Putative glucokinase; transcript regulated upon yeast-hyphal switch; Efg1 regulated; fluconazole-induced; induced in core stress response; colony morphology-related gene regulation by Ssn6; GlcNAc-induced protein |
| P82612 | GPM1 | orf19.903 | Phosphoglycerate mutase; surface protein that binds host complement Factor H and FHL-1; antigenic; fluconazole, or amino acid starvation (3-AT) induced, farnesol-repressed; Hap43, flow model biofilm induced; Spider biofilm repressed |
| P83775 | GRP2 | orf19.4309 | NAD(H)-linked methylglyoxal oxidoreductase involved in regulation of methylglyoxal and pyruvate levels; regulation associated with azole resistance; induced in core stress response or by oxidative stress via Cap1, fluphenazine, benomyl |
| Q59VN4 | HHF1 | orf19.8661 | Putative histone H4; repressed in fkh2 mutant; regulated by Efg1; fluconazole induced; amphotericin B repressed; farnesol regulated; colony morphology-related gene regulation by Ssn6; Hap43-induced; rat catheter and Spider biofilm repressed |
| A0A1D8PNC7 | HSP12 | orf19.4216 | Putative heat shock protein; decreased expression in hyphae; transcription is increased in populations of cells exposed to |

| | | | |
|---|---|---|---|
| | | | fluconazole over multiple generations; overexpression increases resistance to farnesol and azoles |
| O74261 | HSP60 | orf19.717 | Heat shock protein; soluble in hyphae; regulated by Nrg1 and by iron; induced in high iron; heavy metal (cadmium) stress-induced; sumoylation target; protein present in exponential and stationary phase cells; Hap43-repressed |
| P41797 | HSP70 | orf19.4980 | Putative hsp70 chaperone; role in entry into host cells; heat-shock, amphotericin B, cadmium, ketoconazole-induced; surface localized in yeast and hyphae; antigenic in host; farnesol-downregulated in biofilm; Spider biofilm induced |
| P46598 | HSP90 | orf19.6515 | Essential chaperone, regulates several signal transduction pathways and temperature-induced morphogenesis; activated by heat shock, stress; localizes to surface of hyphae, not yeast cells; mediates echinocandin and biofilm azole resistance |
| Q59RB8 | ICL1 | orf19.6844 | Isocitrate lyase; glyoxylate cycle enzyme; required for virulence in mice; induced upon phagocytosis by macrophage; farnesol regulated; Pex5-dependent peroxisomal localization; stationary phase enriched; rat catheter, Spider biofilm induced |
| Q59KV8 | LSP1 | orf19.3149 | Eisosome component with a predicted role in endocytosis; protein present in exponential and stationary growth phase yeast cultures; caspofungin repressed; biofilm induced; fungal-specific (no human/murine homolog); sumoylation target |
| P83778 | MDH1 | orf19.7481 | Mitochondrial malate dehydrogenase; regulated by Mig1, Tup1, white-opaque switch, phagocytosis; induced in high iron; antigenic during murine and human infection; repressed in Spider biofilms by Bcr1, Tec1, Ndt80, Rob1, Brg1 |
| Q5AMP4 | MDH1-1 | orf19.4602 | Predicted malate dehydrogenase precursor; macrophage-induced transcript; protein present in exponential and stationary growth phase yeast cultures; Spider biofilm repressed |
| P82610 | MET6 | orf19.2551 | Essential 5-methyltetrahydropteroyltriglutamate-homocysteine methyltransferase (cobalamin-independent methionine synthase); antigenic in murine/human systemic infection; heat shock, estrogen, GCN-induced; Spider biofilm repressed |
| A0A1D8PSE1 | MLC1 | orf19.2416.1 | Microtubule-dependent localized protein; at Spitzenkorper and cytokinetic ring in hyphae; cell-cycle dependent localization to tip polarisome, bud neck in yeast and pseudohyphae; sumoylation target; rat catheter, Spider biofilm repressed |
| Q5APD2 | MLS1 | orf19.4833 | Malate synthase; glyoxylate cycle enzyme; no mammalian homolog; regulated upon white-opaque switch; phagocytosis, strong oxidative stress induced; stationary phase enriched; flow model biofilm repressed; rat catheter, Spider biofilm induced |
| Q5A470 | orf19.1239 | orf19.8824 | Secreted protein; exogenously expressed protein is a substrate for Kex2 processing in vitro; fluconazole-regulated; Spider biofilm induced |
| Q5ABD5 | orf19.13456 | orf19.6035 | Protein of unknown function; repressed by nitric oxide, induced by hydrogen peroxide |
| A0A1D8PD97 | orf19.5201.1 | orf19.5201.1 | Has domain(s) with predicted ATPase inhibitor activity, role in negative regulation of ATP-dependent activity and mitochondrion localization |
| Q59U89 | orf19.9418 | orf19.9418 | Possible stress protein; increased transcription associated with CDR1 and CDR2 overexpression or fluphenazine treatment; regulated by Sfu1, Nrg1, Tup1; stationary phase enriched protein; Spider biofilm induced |
| A0A1D8PRM7 | PCK1 | orf19.7514 | Phosphoenolpyruvate carboxykinase; glucose, C-source, yeast-hypha, Hap43 regulated; fluconazole, phagocytosis, H2O2, oral candidasis, Spider/rat catheter/flow model biofilm induced; repressed in biofilm by Bcr1, Tec1, Ndt80, Rob1, Brg1 |
| P83779 | PDC11 | orf19.2877 | Pyruvate decarboxylase; antigenic; on hyphal not yeast cell surface; Hap43, Gcn4, Efg1, Efh1, Hsf1 regulated; fluconazole, farnesol induced; amino acid starvation repressed; flow model biofilm induced; Spider biofilm repressed |
| A0A1D8PR99 | PDI1 | orf19.5130 | Putative protein disulfide-isomerase; antigenic in human infection; soluble protein in hyphae; induced by filamentous growth; protein present in exponential and stationary growth phase yeast cultures; flow model biofilm repressed |
| Q5A516 | PET9 | orf19.8545 | Mitochondrial ADP/ATP carrier protein involved in ATP biosynthesis; possible lipid raft component; 3 predicted transmembrane helices; flucytosine induced; ketoconazole-induced; downregulated by Efg1p |
| P83780 | PGI1 | orf19.3888 | Glucose-6-phosphate isomerase; enzyme of glycolysis; antigenic; Efg1-regulated; induced upon adherence to polystyrene; repressed |

| | | | |
|---|---|---|---|
| | | | by phagocytosis, human neutrophils; flow model biofilm induced; rat catheter and Spider biofilm repressed |
| P46273 | PGK1 | orf19.3651 | Phosphoglycerate kinase; localizes to cell wall and cytoplasm; antigenic in murine/human infection; flow model biofilm, Hog1-, Hap43-, GCN-induced; repressed upon phagocytosis; repressed in Spider biofilms by Bcr1, Ndt80, Rob1, Brg1 |
| A0A1D8PJ01 | PMA1 | orf19.5383 | Plasma membrane H(+)-ATPase; highly expressed, comprises 20-40% of total plasma membrane protein; levels increase at stationary phase transition; fluconazole induced; caspofungin repressed; upregulated in RHE model; Spider biofilm repressed |
| A0A1D8PRH0 | PRB1 | orf19.7196 | Putative vacuolar protease; upregulated in the presence of human neutrophils; Spider biofilm induced |
| Q5A5A0 | PRX1 | orf19.5180 | Thioredoxin peroxidase; transcriptionally induced by interaction with macrophage; fluconazole induced; Fkh2p-downregulated; caspofungin repressed; protein present in exponential and stationary growth phase yeast cultures |
| P83782 | QCR2 | orf19.2644 | Ubiquinol-cytochrome-c reductase; antigenic; induced by interaction with macrophage; repressed by nitric oxide; in detergent-resistant membrane fraction (possible lipid raft component); levels decrease in stationary phase; Hap43p-repressed |
| Q5AIB8 | RPL10 | orf19.2935 | Ribosomal protein L10; intron in 5'-UTR; downregulated upon phagocytosis by murine macrophage; transcription regulated by yeast-hypha switch; Spider biofilm repressed |
| Q9UVJ4 | RPL10A | orf19.3465 | Predicted ribosomal protein; downregulated upon phagocytosis by murine macrophages; Hap43-induced; Spider biofilm repressed |
| Q5AJF7 | RPL12 | orf19.1635 | Ribosomal protein L12, 60S ribosomal subunit; downregulated by human whole blood or polymorphonuclear cells; genes encoding cytoplasmic ribosomal subunits are downregulated upon phagocytosis by macrophage; Tbf1p-activated; Hap43p-induced |
| A0A1D8PK43 | RPL18 | orf19.5982 | Predicted ribosomal protein; Plc1p-regulated, Tbf1-activated; repressed upon phagocytosis by murine macrophage; Hap43p-induced; Spider biofilm repressed |
| A0A1D8PK40 | RPL19A | orf19.5904 | Ribosomal protein L19; repressed upon phagocytosis by murine macrophages; Hap43-induced gene; Spider biofilm repressed |
| A0A1D8PH21 | RPL36 | orf19.827.1 | Ribosomal protein L39; transcript induced upon germ tube formation; colony morphology-related gene regulation by Ssn6; Hap43-induced |
| A0A1D8PCX8 | RPL6 | orf19.3003.1 | Ortholog of S. cerevisiae ribosomal subunit, Rpl6B; transposon mutation affects filamentous growth; translation-related genes are downregulated upon phagocytosis by murine macrophage; Hap43-induced; Spider biofilm repressed |
| A0A1D8PQS0 | RPP0 | orf19.7015 | Putative ribosomal protein; antigenic in mouse; repressed upon phagocytosis by murine macrophage; induced by Tbf1; overlaps orf19.7014; Spider biofilm repressed |
| O42817 | RPS0 | orf19.6975 | Ribosome-associated protein; antigenic in mice; complements S. cerevisiae yst1 yst2 mutant; similar to laminin receptor; predicted S/T phosphorylation, N-glycosylation, myristoylation, Hap43-, Gcn4-regulated; Spider biofilm repressed |
| P40910 | RPS1 | orf19.3002 | Putative ribosomal protein 10 of the 40S subunit; elicits host antibody response during infection; transcript induced during active growth; Spider biofilm repressed |
| A0A1D8PI15 | RPS10 | orf19.2179.2 | Ribosomal protein S10; downregulated in the presence of human whole blood or PMNs; Spider biofilm repressed |
| Q5ADQ6 | RPS12 | orf19.6785 | Acidic ribosomal protein S12; regulated by Gcn4, activated by Tbf1; repressed by amino acid starvation (3-AT); protein abundance is affected by URA3 expression in CAI-4 strain background; sumoylation target; Spider biofilm repressed |
| A0A1D8PDT3 | RPS14B | orf19.6265.1 | Putative ribosomal protein; repressed upon phagocytosis by murine macrophage; transcript positively regulated by Tbf1; Spider biofilm repressed |
| A0A1D8PQQ5 | RPS18 | orf19.7018 | Predicted ribosomal protein; repressed upon phagocytosis by murine macrophage; repressed by nitric oxide; Hap43-induced; Spider biofilm repressed |
| A0A1D8PK61 | RPS19A | orf19.5996.1 | Putative ribosomal protein S19; protein level decreases in stationary phase cultures; Spider biofilm repressed |
| A0A1D8PCG7 | RPS21B | orf19.3325.3 | Ribosomal protein S21; regulated by Nrg1, Tup1; colony morphology-related gene regulation by Ssn6; positively regulated by Tbf1, Hap43; Spider biofilm repressed |
| A0A1D8PTI7 | RPS27 | orf19.6286.2 | Putative ribosomal protein; repressed upon phagocytosis by murine macrophage; Spider biofilm repressed |

| | | | |
|---|---|---|---|
| Q5AG43 | RPS5 | orf19.4336 | Ribosomal protein S5; macrophage/pseudohyphal-induced after 16 h; downregulated upon phagocytosis by murine macrophage; Hap43-induced; Spider biofilm repressed |
| A0A1D8PGY8 | RPS9B | orf19.838.1 | Predicted ribosomal protein; repressed upon phagocytosis by murine macrophage; transcript possibly regulated upon hyphal formation; Spider biofilm repressed |
| A0A1D8PF90 | SCP160 | orf19.5281 | Predicted essential RNA-binding G protein; ortholog an effector of mating response pathway in S. cerevisiae; mainly associated with nuclear envelope and ER; flow model and Spider biofilm repressed |
| Q5AIA6 | SNZ1 | orf19.2947 | Stationary phase protein; vitamin B synthesis; induced byyeast-hypha switch, 3-AT or in azole-resistant strain overexpressing MDR1; soluble in hyphae; regulated by Gcn4, macrophage; Spider biofilm induced; rat catheter biofilm repressed |
| Q5A8Z4 | SOD2 | orf19.3340 | Mitochondrial Mn-containing superoxide dismutase; protection against oxidative stress; homotetramer active; N-terminal 34 amino acids removed on mitochondrial import; H2O2-induced via Cap1p; Hap43p-, alkaline-downregulated, farnesol-induced |
| A0A1D8PQH5 | SOD3 | orf19.7111.2 | Cytosolic manganese-containing superoxide dismutase; protects against oxidative stress; repressed by ciclopirox olamine, induced during stationary phase when SOD1 expression is low; Hap43-repressed; Spider and flow model biofilm induced |
| Q5A397 | SSB1 | orf19.6367 | HSP70 family protein chaperone, possibly essential; involved in caspofungin tolerance via phosphorylation state; target of the Ptc2p phosphatase; expressed at yeast cell surface, not hyphae; antigenic in human/mouse infection |
| Q59LQ6 | SUI1 | orf19.8867 | Putative translation initiation factor; flucytosine induced; genes encoding ribosomal subunits, translation factors, and tRNA synthetases are downregulated upon phagocytosis by murine macrophage |
| Q5ADM7 | TDH3 | orf19.14106 | NAD-linked glyceraldehyde-3-phosphate dehydrogenase; binds fibronectin, laminin; at cell surface; antigenic in infection; farnesol-repressed; stationary phase-enriched; GlcNAc-induced; flow model biofilm induced; Spider biofilm repressed |
| Q5A1M1 | TFS1 | orf19.1974 | Putative carboxypeptidase y inhibitor; transcript regulated upon yeast-hypha switch; colony morphology-related gene regulation by Ssn6 |
| A0A1D8PL02 | TIM11 | orf19.5660.1 | Ortholog(s) have proton-transporting ATP synthase activity, rotational mechanism, structural molecule activity and role in cristae formation, protein-containing complex assembly, proton motive force-driven ATP synthesis |
| Q5A750 | TKL1 | orf19.5112 | Putative transketolase; localizes to surface of yeast cells, not hyphae; soluble protein in hyphae; transcript regulated by Nrg1, Mig1, and Tup1; antigenic in human or murine infection; possibly essential (by UAU1 method) |
| A0A1D8PC97 | TUB2 | orf19.6034 | Beta-tubulin; functional homolog of ScTub2; overproduction makes S. cerevisiae inviable; has two introns; GlcNAc, hypha fluconazole-induced; slow growth, ectopic expression increases white-to opaque switch; rat catheter biofilm repressed |
| Q5A109 | UBI3 | orf19.3087 | Fusion of ubiquitin with the S34 protein of the small ribosomal subunit; mRNA decreases upon heat shock, appears to be degraded; functional homolog of S. cerevisiae RPS31; Hap43-induced; Spider biofilm repressed |

**Table S2. *C. albicans* proteome of EVs isolated from rat catheters.**

| UniProt ID# | Gene name | Gene ORF# | Description | *in vitro* EVs | *in vitro* ECM | *in vivo* EVs |
|---|---|---|---|---|---|---|
| Q5AP53 | CBK1 | orf19.4909 | Ser/Thr kinase of cell wall integrity pathway; mutants show abnormal morphology and aggregation; Mob2p associated; required for wild-type hyphal growth and transcriptional regulation of cell-wall-associated genes | | | **x** |
| Q59MA9 | CLU1 | orf19.51 | Ortholog(s) have RNA binding activity | | **x** | **x** |
| A0A1D8PIK8 | COE1 | orf19.1371 | Mitochondrial protein required for expression of respiratory chain complex IV (cytochrome c oxidase) | | | **x** |
| Q5A4Z0 | CSF1 | orf19.956 | Ortholog(s) have role in fermentation, phospholipid homeostasis, protein maturation | | | **x** |
| A0A1D8PFU3 | DOA4 | orf19.7207 | Ortholog of S. cerevisiae Doa4;, a ubiquitin hydrolase involved in recycling ubiquitin from proteasome-bound ubiquitinated intermediates; oxidative stress-induced via Cap1; mutants are viable | | | **x** |
| Q59QC2 | EAF6 | orf19.396 | Subunit of the NuA4 histone acetyltransferase complex; Hap43-repressed; Spider biofilm repressed | | | **x** |
| A0A1D8PTY4 | ELF1 | orf19.7332 | Putative mRNA export protein; Walker A and B (ATP/GTP binding) motifs; required for wild-type morphology, growth; expressed in hyphal, pseudohyphal, and yeast form; Hap43-induced; Spider and flow model biofilm induced | **x** | **x** | **x** |
| Q5AIA1 | EXG2 | orf19.2952 | GPI-anchored cell wall protein, similar to S. cerevisiae exo-1,3-beta-glucosidase Exg2p; predicted Kex2p substrate; induced during cell wall regeneration; possibly an essential gene, disruptants not obtained by UAU1 method; Hap43p-repressed | **x** | **x** | **x** |
| A0A1D8PTM6 | FGR32 | orf19.5933 | Protein of unknown function; transcript induced by benomyl treatment | | | **x** |
| A0A1D8PJ45 | FLC1 | orf19.2501 | Protein involved in heme uptake; putative FAD transporter, similar to S. cerevisiae Flc1; regulated by iron; macrophage-induced; mutant defective in filamentous growth; Spider biofilm induced | **x** | **x** | **x** |
| Q5ADV5 | FMP38 | orf19.6736 | Protein required for mitochondrial ribosome small subunit biogenesis; role in maturation of SSU-rRNA; Spider biofilm induced | | | **x** |
| Q5AG77 | GAP1 | orf19.4304 | Amino acid permease; antigenic in human/mouse; 10-12 transmembrane regions; regulated by nitrogen source; alkaline, GlcNAc, phagocytosis induced; WT virulence in mice; Spider and flow model biofilm induced | | | **x** |
| Q5ADR6 | GCD2 | orf19.6776 | Putative translation initiation factor; genes encoding ribosomal subunits, translation factors, and tRNA synthetases are downregulated upon phagocytosis by murine macrophage | | | **x** |
| Q59NC4 | GLY1 | orf19.986 | L-threonine aldolase; complements glycine auxotrophy of S. cerevisiae shm1 shm2 gly1-1 triple mutant; macrophage/pseudohyphal-induced; the GLY1 locus has an RFLP and is triploid in strain SGY269; flow model biofilm induced | | | **x** |

| | | | | | | |
|---|---|---|---|---|---|---|
| A0A1D8PJG1 | GPA2 | orf19.1621 | G-protein alpha subunit; regulates filamentous growth, copper resistance; involved in cAMP-mediated glucose signaling; reports differ on role in cAMP-PKA pathway, MAP kinase cascade; Gpr1 C terminus binds Gpa2; regulates HWP1 and ECE1 | x | x | x |
| Q5AAL9 | IFF4 | orf19.7472 | Adhesin-like cell surface protein; putative GPI-anchor; null mutant germ tubes show decreased adhesion to plastic substrate; mutants are viable; Hap43-repressed gene | | | x |
| A0A1D8PIJ7 | IFO2 | orf19.1765 | Secreted protein; fluconazole-induced | x | | x |
| Q59S66 | IPL1 | orf19.3474 | Putative Aurora kinase; Hap43-induced; induced during planktonic growth; possibly an essential gene, disruptants not obtained by UAU1 method | | | x |
| A0A1D8PJ26 | KGD1 | orf19.6165 | Putative 2-oxoglutarate dehydrogenase; regulated by Efg1 under yeast but not hyphal growth conditions; transcript induced in an RHE model of oral candidiasis; stationary phase enriched protein; Hap43-repressed; rat catheter biofilm induced | x | x | x |
| A0A1D8PFW9 | NCR1 | orf19.7242 | Putative vacuolar membrane protein; predicted role in sphingolipid metabolism; transcript regulated by Nrg1 and Mig1; induced by prostaglandins | | x | x |
| A0A1D8PQD3 | orf19.1087 | orf19.1087 | Ortholog of C. dubliniensis CD36 : Cd36_64620, C. parapsilosis CDC317 : CPAR2_601130, Candida tropicalis MYA-3404 : CTRG_02757 and Candida albicans WO-1 : CAWG_04949 | | | x |
| A0A1D8PM19 | orf19.1426 | orf19.1426 | Ortholog of S. cerevisiae Skg6; localizes to the cell cortex, cell bud neck, cell bud tip, incipient cellular bud site, and is membrane-bound; Spider biofilm induced | | | x |
| Q5ALV4 | orf19.1473 | orf19.1473 | 2-hydroxyacid dehydrogenase domain-containing protein; Hap43-repressed gene; induced by alpha pheromone in SpiderM medium | | | x |
| A0A1D8PJD5 | orf19.1634 | orf19.1634 | Has domain(s) with predicted fatty-acyl-CoA binding activity | | | x |
| A0A1D8PE20 | orf19.4423 | orf19.4423 | Putative glucosyltransferase; localized to the mitochondrial membrane | | | x |
| Q5AMS7 | orf19.4569 | orf19.4569 | Ortholog of C. dubliniensis CD36 : Cd36_42100, Debaryomyces hansenii CBS767 : DEHA2C14850g, Pichia stipitis Pignal : PICST_52615 and Candida tropicalis MYA-3404 : CTRG_00173 | | | x |
| Q5AMN1 | orf19.4615 | orf19.4615 | Ortholog(s) have histone deacetylase activity | | | x |
| Q5A1L3 | orf19.588 | orf19.588 | Ortholog(s) have role in aerobic respiration and mitochondrial intermembrane space localization | | | x |
| Q5ANH0 | orf19.5933 | orf19.5933 | Protein of unknown function; transcript induced by benomyl treatment | | | x |
| Q5AAN5 | orf19.6276 | orf19.6276 | Putative nucleolar protein; constituent of pre-60S ribosomal particles; Hap43-induced; repressed by prostaglandins | | | x |
| A0A1D8PU23 | orf19.7554 | orf19.7554 | Transporter; similar to the Sit1 siderophore transporter; induced by nitric oxide independent of Yhb1; repressed during chlamydospore formation in C. albicans and C. dubliniensis; rat catheter biofilm repressed | | | x |

| | | | | | | |
|---|---|---|---|---|---|---|
| A0A1D8PQ11 | PEX25 | orf19.5575 | Putative peripheral peroxisomal membrane peroxin; required for regulating peroxisome size and maintenance; Spider biofilm induced | | | x |
| Q59X73 | PHH1 | orf19.604 | Has domain(s) with predicted FAD binding activity | | | x |
| A0A1D8PP34 | PPR1 | orf19.3986 | Transcription factor with zinc cluster DNA-binding motif involved in regulation of purine catabolism; has similarity to S. cerevisiae Ppr1p, which is a transcription factor involved in the regulation of uracil biosynthesis genes | | | x |
| Q59SI1 | RLP24 | orf19.4191 | Putative ribosomal protein; Hap43-induced; essential gene; heterozygous mutation confers hypersensitivity to 5-fluorocytosine (5-FC), 5-fluorouracil (5-FU), and tubercidin (7-deazaadenosine); Spider biofilm induced | | | x |
| Q5ALV6 | RPS26A | orf19.1470 | Ribosomal protein; regulated by Nrg1, Tup1; repressed upon phagocytosis by murine macrophage; alternatively spliced intron in 5'-UTR; Spider biofilm repressed | | x | x |
| A0A1D8PIJ2 | RRP15 | orf19.563 | Putative nucleolar protein; constituent of pre-60S ribosomal particles; Hap43-induced; repressed by prostaglandins | | | x |
| A0A1D8PUA5 | SEO13 | orf19.7666 | Predicted membrane transporter, member of the anion:cation symporter (ACS) family, major facilitator superfamily (MFS) | | | x |
| Q5A3K7 | SER33 | orf19.5263 | Predicted enzyme of amino acid biosynthesis; Gcn4p-regulated; upregulated in biofilm; protein present in exponential and stationary growth phase yeast cultures; S. cerevisiae ortholog is Gcn4p regulated ( | x | x | x |
| Q59Q34 | SPF1 | orf19.30 | P-type calcium-transporting ATPase, involved in control of calcium homeostasis, response to ER stress, hyphal growth, biofilm formation and virulence | | x | x |
| Q5AMR1 | TFG1 | orf19.4585 | Protein similar to S. cerevisiae Tfg1p, which is part of transcription factor TFIIF; transposon mutation affects filamentous growth; possibly an essential gene, disruptants not obtained by UAU1 method | | | x |
| A0A1D8PDM3 | TMT1 | orf19.2468 | Ortholog(s) have trans-aconitate 3-methyltransferase activity, role in cellular response to amino acid starvation and cytosol localization | | | x |
| Q5A2B2 | TRF4 | orf19.429 | Putative non-canonical poly(A) polymerase; repressed by nitric oxide; Spider biofilm induce | | | x |
| Q5A2U9 | VMA5 | orf19.2166 | Putative vacuolar H(+)-ATPase; plasma membrane localized; rat catheter biofilm repressed | x | x | x |
| Q5A314 | YFW4 | orf19.6449 | Ortholog of C. dubliniensis CD36 : Cd36_34310 | | | x |

EV - extracellular vesicle, ECM - extracellular matrix, X indicates presence

**Table S3. Unique cerebroside biomarkers identified in *C. albicans* EVs isolated from rat catheters.**

| Annotation name | Lipid class | MS mode | *m/z* | Molecular formula | Retention time [min] | control uninfected serum | infected input | infected output | in vitro Candida EVs |
|---|---|---|---|---|---|---|---|---|---|
| HexCer 28:1 [M+Ac-H]- | HexCer | negative | 748.5227 | C39O12N1H74 | 1.6935 | <LOD | <LOD | 13698.0 | 1196.5 |
| HexCer 28:2 [M+Ac-H]- | HexCer | negative | 674.4865 | C36O10N1H68 | 1.8051 | <LOD | <LOD | 5084.5 | 94.4 |
| HexCer 31:1 [M+Ac-H]- | HexCer | negative | 672.4692 | C36O10N1H66 | 1.6753 | <LOD | <LOD | 5278.5 | 2957.2 |
| HexCer 37:3 [M+H]+ | HexCer | positive | 738.5885 | C43O8N1H80 | 2.4676 | <LOD | 2748.7 | 4871.8 | 55656.7 |

<LOD is below limit of detection; values are arbitrary units [AU]

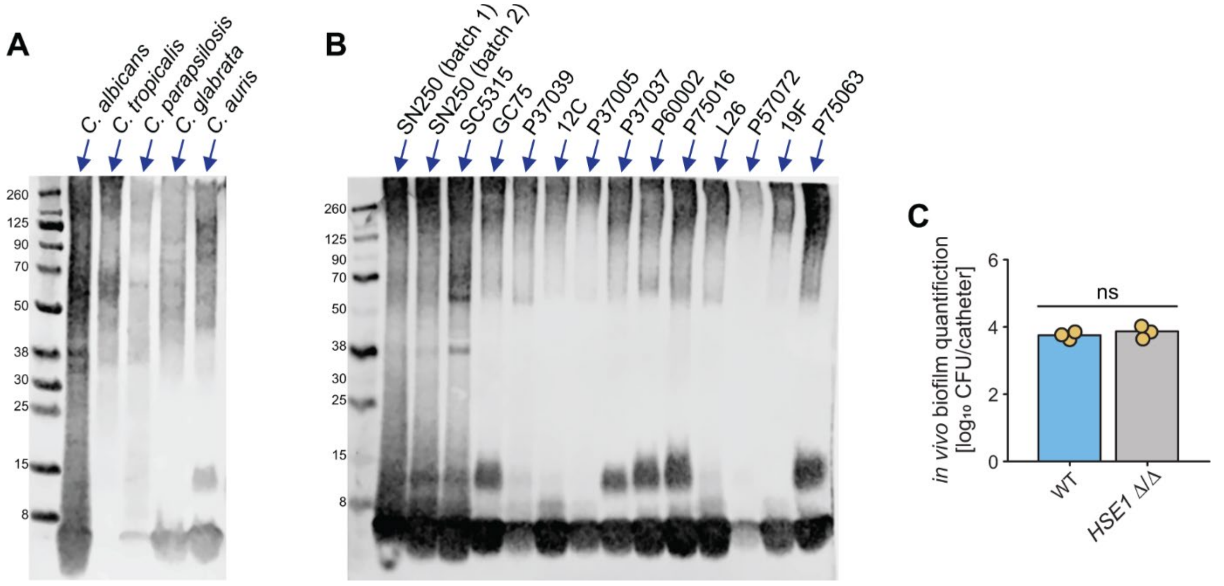


**Figure S1. Isolation and characterization of *Candida* EVs from an in vivo vascular catheter biofilm.** (**A**) Western blot-based detection of EVs isolated from common biofilm-forming Candida species by rabbit polyclonal anti-*C. albicans* EV antibodies. Whole purified EVs were prepared from in vitro biofilms, denatured and run on SDS-Page gels prior to western blotting with anti-EV polyclonal rabbit antibody. Binding was shown in all species tested including *C. albicans* (lane 1), *C. tropicalis* (lane 2), *C. parapsilosis* (lane 3), *C. glabrata* (lane 4), and *C. auris* (lane 5). (**B**) Western blot-based detection of EVs isolated from different clinical isolates of *C. albicans* by rabbit polyclonal anti-*C. albicans* EV antibodies. Whole purified EV preparations were prepared from in vitro biofilms of clinical isolates and were denatured and run on SDS-Page gels prior to western blotting with anti-EV polyclonal rabbit antibody. Binding was shown in all clinical isolates tested including SN250 (lane 1 and 2), SC5315 (lane 3), GC75 (land 4), P37039 (lane 5), 12C (lane 6), P37005 (lane 7), P37037 (lane 8), P60002 (lane 9), P75016 (lane 10), L26 (lane 11), P570516 (lane 12), 19F (lane 13), and P75063 (lane 14 ). (**C**) WT and hse1ΔΔ ESCRT mutant produce similar viable biofilm burden in the in vivo rat vascular catheter model. The viable *C. albicans* burden from 48 h biofilms in the rat catheter model. Each dot is an independent biological replicate. Data are presented as the mean ± SD (bars); *n* = 3 rats per strain. P = non-significant, student's test.

**Supplemental Methods**

Strains and culture conditions. *Candida* isolates were cultured and maintained as previously described. Strains utilized included *Candida albicans* SN250 hseΔΔ, *C. albicans SC5314, C. albicans GC75, C. albicans P37039, C. albicans 12C, C. albicans P37037, C. albicans P37005, C. albicans P60002, C. albicans P75016, C. albicans L26, C. albicans P57072, C. albicans 19F, C. albicans P75063, C tropicalis CAY2597, C. parapsilosis CLIB214, C. glabrata ATCC2001,* and *C. auris B11804.* Briefly, strains were plated on YPD-agar plates. Single colonies were picked and grown overnight in 3 mL of liquid YPD media at 30ºC and used for downstream in vivo and in vitro applications. Small scale in vitro biofilms were plated as previously described in six-well plates in RPMI-MOPS at 37°C [1]. Large scale biofilms for concentrated purified EVs are described below.

Large scale EV purification for antibody production. Purified and concentrated SN250 *Candida albicans* biofilm EVs were generated using methods described previously [1]. Briefly, biofilms were grown in polystyrene roller bottles incubated at 37°C in RPMI+MOPS for 48 hours. Biofilm supernatant was collected and filtered, and concentrated via Hydrosart 30kDa cut-off membrane using a Viavflow 200 unit (Sartorius AG) and then cellular debris was removed with a one-hour spin at 10,000 x g. After debris-removal supernatants were spun at 100,000 x g for 1.5 hours at 4°C to pellet EVs. Pelleted EVs were then size selected with SEC chromatography on qEV/35nm column (Izon Sciences) and stored at 4°C for further analysis and downstream application.

Polyclonal rabbit IgG antibody generation and desthiobiotynlation. Antibodies were produced by ThermoFisher Scientific using our supplied purified and concentrated 48-hour biofilm EVs as the antigen. The standard ThermoFisher Scientific 90-day rabbit protocol was used. On Day 0 rabbits underwent a control bleed with 5mL of pre-immune serum collected for downstream analysis. Rabbits underwent primary immunization with EVs emulsified in Freund's complete adjuvant and subcutaneous inoculated into four sites on Day 1, with booster inoculations on Day 14, Day 28, and Day 56. Rabbits underwent serum collection on Day 28, Day 56, and Day 72. Day 72 bleeds were used for antibody production and purification. Preliminary bleeds were assayed via western blot to assess reactivity compared to initial rabbit serum. Antibodies from day 72 rabbit serum were purified using A/G purification with Piere Protein A/G agarose beads using standard column procedure (ThermoFisher Scientific). Briefly, column was packed with protein A/G resin slurry, and equilibrated with IgG Binding Buffer (ThermoFisher Scientific). Rabbit serum was diluted with binding buffer and placed on the column. The column was then washed again with binding buffer. Antibodies were eluted with ThermoFisher IgG Elution Buffer (ThermoFisher Scientific) and used for downstream experiments. Purified antibodies were tested via western blotting for reactivity against purified *Candida* EVs and used for downstream experiments.

Purified polyclonal anti-CA EV antibody (at 1mg/ML concentration) was reacted with EZ-link™ Sulfo-NHS-LC desthiobiotin (ThermoFisher Scientific) resuspended in DMSO as described in the EZ-Link Desthiobiotinylation and Pull-Down Kit. Briefly, 10uL of resuspended reagent was reacted with 1 mL of anti-EV antibody for sixty minutes at RT with end over end rotation. Reaction was desalted and excess desthiobiotin was removed with Zeba 5mL 7K desalting column (ThermoFisher Scientific). Modified antibody was stored at 4C for downstream applications.

In vivo catheter model and sample collection. Rat catheter model was performed as described previously[2]. Using sterile surgical technique, polyethylene catheters were inserted into the internal jugular vein approximately 2 cm in caesarean-derived Sprague-Dawley 325-350 gram male rats (Charles River Laboratories). Twenty-four hours after placement, the catheters were instilled with

1 x $10^6$ cells/mL. After a 6 hour dwell, the catheters were flushed with saline. After an additional 48 hours of incubation, blood was removed from the catheters for processing. Three rats were utilized for each vesicle isolation and biofilm quantitation experiment. For biofilm SEM and quantitative culture, the distal 1 cm catheter segment were sectioned into 2mm segments for SEM or quantitative culture.

EV immunoprecipitation. Desthiobiotinylated polyclonal rabbit IgG anti EV antibodies were pre-conjugated to Dynabeads™ M-280 Streptavidin beads (ThermoFisher Scientific) for one hour at room temperature and then washed three times in Dulbecco's phosphate buffered saline (ThermoFisher Scientific) with 0.1% BSA (Sigma Aldrich) to remove excess antibody. Antibody coated beads were then added to samples and allowed to incubate at room temperature with end over end gentle rotation for 1 hour. Then using a DynaMag2™ magnetic rack (ThermoFisher Scientific), beads were washed in sterile PBS four times to remove non-binding elements. EVs were eluted from beads with biotin elution buffer (4mM biotin, 20mM Tris, 50mM NaCl) (ThermoFisher Scientific) with incubation at 37C for 30 minutes with gentle mixing and used in downstream analysis.

Western Blotting. Protein samples were boiled at 95C for 10 minutes in 4x protein sample loading buffer (Li-COR Biotechnology, Lincoln NE US). Samples were loaded onto precast polyacrylamide 15 well Bolt 4-12% gradient Bis-Tris gels (ThermoFisher Scientific) and run for approximately 24 minutes in MES buffer. Chameleon® Duo Pre-stained Protein Ladder was used for size determination (Li-COR Biotechnology). Gels were then washed for 5 minutes on in 20% ethanol and transferred to nitrocellulose membranes using the iBlot 2 semi-dry transfer device (ThermoFisher Scientific). Nitrocellulose membranes were blocked using LiCor Intercept® Blocking Buffer (TBS) (Li-COR Biotechnology) for 1 hour at room temperature with gentle rocking. Purified rabbit EV antibody (ThermoFisher Scientific, custom product) was added to Intercept® T20 TBS Antibody Diluent (Li-COR Biotechnology) at a ratio of 1:200 and blots were incubated at room temperature with gentle rocking for one hour at room temperature. Blots were washed 3 x 5 minutes in TBS-Tween20 and then incubated with secondary antibody IRDye ® 800CW donkey-anti-rabbit at 1:10,000 dilution. Blots were imaged using the LiCor Odyessy Fc Western Blot Imager running Image Studio™ software (Li-COR Biotechnology).

EV particle analysis. Exosomes were quantified using a combination of imaging flow cytometry (IFC) and nanoparticle tracking analysis (NTA). Prior to IFC analysis, samples were stained with carboxyfluorescein succinimidyl ester (CSFE) and 1,1'-dioctadecyl-3,3,3',3'-tetramethylindocarbocyanine perchlorate (DiI) at 37°C for 90 min. Excessive dye particles were removed from stained vesicles using illustra microspin G-50 columns (GE Healthcare). All samples were analyzed on the ImageStreamX Mk II flow cytometry system ( Amnis Corporation) at ×60 magnification, with default low flow rate/high sensitivity using the INSPIRE software.

Prior to NTA testing, EV samples were diluted in PBS to a final volume of 1 ml and pretested to obtain an ideal 30-100 particles per frame rate using a NanoSight NS300 system (Malvern). The following settings were applied: camera level was increased to 16 and camera gain to 2 until tested images were optimized and nanoparticles were distinctly visible without exceeding particle signal saturation. Each measurement consisted of five 1-min videos with a delay of 5 s between sample introduction and the start of the first measurement. For detection threshold analysis the counts were limited to 10-100 red crosses and no more than 5-7 blue crosses. Acquired data were analyzed using the NanoSight Software NTA 3.4 Build 3.4.003. At least 1000 events in total was tracked per sample in order to minimize data skewing based on single large particles.

In vivo proteinase K vesicle treatment. Purified in vivo vesicles were diluted 1:100 in PBS in 1.5 mL Eppendorf tubes. Experimental samples were treated with 16 IU of proteinase K per 1 mL of diluted EVs (NEB Biosciences) at 37C. Samples were then immunoprecipitated per standard protocol with desthiobiotynlated anti-EV antibody and streptavidin coated beads. After elution with from the beads, samples, and untreated controls were run on SEC column to remove low molecular weight digested proteins and samples were then subjected to western blotting with anti-EV antibody and analyzed on NanoSight NS300 as described below.

Cryo-electron microscopy. 200-mesh lacey carbon grids were glow-discharged for 30 seconds in a Pelco easiGlow glow-discharger at 15mA with a chamber pressure of 0.24 mBar. Plunge freezing occurred in an FEI Vitrobot Mark IV cryo plunge freezing robot. Four µl of sample were the pipetted onto the grid, blotted for 5 seconds with a blotting pressure of 1, and plunge-frozen into liquid ethane. Grids were transferred into liquid nitrogen for storage, loaded into a Gatan 626.5 cryo transfer holder cooled to -180 ºC, and imaged in a JEOL JEM1230 W-emission TEM at 120kV. Data was collected using Gatan Digital Micrograph software connected to a Gatan Orius SC1000 CCD Camera, Model 831.

Mass spectrometry-based proteomics. EVs bound to anti-EV beads were lysed with 50µl of 3% SDS in 30mM Tris HCl pH 8.0 while incubating for 10 minutes at 56°C. Subsequently 50µl of MilliQ water was added (1.5% SDS and 10mM Tris final concentration), liquid transferred from beads to new 1.5ml tube and samples were sonicated for 2 minutes in the sonication bath. 100µl of unenriched EVs in serum depleted samples (treated with Cibracon Blue and rat-serum antibodies) were lysed with 12µl addition of 10% SDS (1% final) and sonicated for 2 minutes in the sonication bath. Finally, to 100µl of rat catheter flush 12µl of 10% SDS (1% final) was added and samples were sonicated for 2 minutes in the sonication bath. Proteins released from these sample groups were precipitated with 10% TCA (vol:vol) in 50% acetone (vol:vol) for 30 minutes on ice. Spun for 10 minutes at room temperature with max speed (16,000xg) and generated pellets were washed twice with cold acetone. Generated protein pellets were re-solubilized and denatured in 15µl of 8M Urea in 50mM $NH_4HCO_3$ (pH8.5). Subsequently diluted to 60µl for reduction step with: 2.5µl of 25mM DTT and 42.5µl of 25mM $NH_4HCO_3$ (pH8.5). Incubated at 56°C for 15 minutes, cooled on ice to room temperature then 3µl of 55mM CAA (chloroacetaminde) was added for alkylation and incubated in darkness at room temperature for 15 minutes. Reaction was quenched by adding 8µl of 25mM DTT. Finally, 5µl of Trypsin/LysC solution [100ng/µl 1:1 *Trypsin* (Promega) and *LysC* (FujiFilm) mix in 25mM $NH_4HCO_3$] and 24µl of 25mM $NH_4HCO_3$ (pH8.5) was added to pre-cleared serum and anti-EVs enriched samples while 29µl of Trypsin/LysC solution was added to catheter flush samples for 100µl final volume, digestion proceeded o/n at 37°C. Reaction was terminated by acidification with 2.5% TFA [Trifluoroacetic Acid] to 0.3% final.

Digests were desalted using Pierce™ C18 SPE pipette tips (100µl volume) per manufacturer protocol and eluted in 20µl of 70/30/0.1% ACN/$H_2O$/TFA. Dried to completion in the speed-vac and finally reconstituted in either 15µl for pre-cleared serum and anti-EVs enriched samples or 25 µl for catheter flush samples with 0.1% formic acid containing 2% acetonitrile. Peptides were analyzed by nanoLC-MS/MS using the Agilent 1100 nanoflow system (Agilent) connected to hybrid linear ion trap-orbitrap mass spectrometer (LTQ-Orbitrap Elite™, Thermo Fisher Scientific) equipped with an EASY-Spray™ electrospray source (held at constant 45°C). Chromatography of peptides prior to mass spectral analysis was accomplished using capillary emitter column (PepMap® C18, 3µM, 100Å, 150x0.075mm, Thermo Fisher Scientific) onto which 3µl of extracted peptides was automatically loaded. NanoHPLC system delivered solvents A: 0.1% (v/v) formic acid , and B: 99.9% (v/v) acetonitrile, 0.1% (v/v) formic acid at 0.50 µL/min to load the peptides (over a 30 minute period) and 0.3µl/min to elute peptides directly into the nano-electrospray with

gradual gradient from 0% (v/v) B to 30% (v/v) B over 80 minutes and concluded with 5 minute fast gradient from 30% (v/v) B to 50% (v/v) B at which time a 5 minute flash-out from 50-95% (v/v) B took place. As peptides eluted from the HPLC-column/electrospray source survey MS scans were acquired in the Orbitrap with a resolution of 120,000 followed by CID-type MS/MS fragmentation of 30 most intense peptides detected in the MS1 scan from 350 to 1800 m/z; redundancy was limited by dynamic exclusion.

Elite acquired MS/MS data files were converted to mgf file format using MSConvert (ProteoWizard: Open Source Software for Rapid Proteomics Tools Development) and searched using Mascot (Matrix Science, London, UK; version 3.0.0). Mascot was set up to search the UniProtKB *Rattus norvegicus* (UP000002494, 04-05-2021 download; 31,564 entries) and *Candida albicans SC5314* (UP000000559, 10-18-2024 download; 6,036 entries) reference databases along with general contaminants added (GPM cRAP database; cRAP_20211123), assuming the digestion enzyme trypsin. Mascot was searched with a fragment ion mass tolerance of 0.60 Da and a parent ion tolerance of 10.0 PPM. Carbamidomethylation of cysteine was specified in Mascot as fixed modifications. Deamidation of asparagine and glutamine and oxidation of methionine were specified in Mascot as variable modifications. Scaffold (version 5.0.1, Proteome Software Inc., Portland, OR) was used to validate MS/MS based peptide and protein identifications. Peptide identifications were accepted if they could be established at greater than 29.0% probability to achieve an FDR less than 1.0% by the Scaffold Local FDR algorithm. Protein identifications were accepted if they could be established at greater than 99.0% probability to achieve an FDR less than 1.0% and contained at least 2 identified peptides. Protein probabilities were assigned by the Protein Prophet algorithm [3]. Proteins that contained similar peptides and could not be differentiated based on MS/MS analysis alone were grouped to satisfy the principles of parsimony. Proteins sharing significant peptide evidence were grouped into clusters.

The mass spectrometry proteomics data have been deposited to the ProteomeXchange Consortium via the PRIDE partner repository with the dataset accession: PXD076431

Targeted metabolomics instrumental analysis and data processing. Metabolite extracts were analyzed by LC-MS/MS. 4 µL of each sample was injected and separated by UHPLC using a Nexera UHPLC system (DGU-405 degasser unit, LC40DX3 solvent delivery system, SIL-40CX3 autosampler, CBM-40 system controller, CTO-40C column oven; Shimadzu). Separation was achieved by normal-phase liquid chromatography using an Acquity Premier CSH C18 1.7 µm VanGuard 2.1 x 50 mm (186009460, Waters). Separation was achieved using a 5-minute multi-phase linear gradient of the following buffers: Buffer A) 6:4 acetonitrile: H2O (v/v) 1 mM ammonium acetate, B: 9:1 isopropyl alcohol: acetonitrile with 1 mM ammonium acetate. Samples were ionized using an Optimus Turbo V + Dual TIS ion source (Sciex) configured for APCI and were analyzed using an X500R mass spectrometer (Sciex). Samples were analyzed in positive ionization mode and were acquired using a high resolution multiple reaction monitoring method.

LC-MS/MS data processing and ion annotation was performed according to accepted protocols for mass spectrometry data processing and feature annotation. Briefly, annotation was based on matching of chromatographic retention times, MS1 values from detected features, and the resulting MS2 fragment ions to expected retention time ranges for measured purified metabolite standards, as well as observed and literature reported precursor and fragment masses.

Untargeted LC-MS lipidomics instrumental analysis and data processing. Metabolite extracts were analyzed by LC-MS/MS. 4 µL of each sample was injected and separated by UHPLC using

a Nexera UHPLC system (DGU-405 degasser unit, LC40DX3 solvent delivery system, SIL-40CX3 auto sampler, CBM-40 system controller, CTO-40C column oven; Shimadzu). Separation was achieved by reverse-phase liquid chromatography using an Acquity Premier CSH C18 column with VanGuard FIT (1.7 µm, 2.1 mm X 50 mm; 186009463, Waters). Separation was achieved using a 5-minute multi-phase linear gradient of the following buffers: Buffer A) 6:4 acetonitrile: H2O (v/v) 1 mM ammonium acetate, B: 9:1 isopropyl alcohol: acetonitrile (9:1, v/v) with 1 mM ammonium acetate. Samples were ionized using an Optimus Turbo V + Dual TIS ion source (Sciex) and were analyzed using an X500R mass spectrometer (Sciex). Samples were analyzed in positive and negative ionization mode and were acquired using a high-resolution data dependent acquisition method.

LC-MS/MS data processing and ion annotation was performed according to accepted protocols for mass spectrometry data processing and feature annotation. Briefly, annotation was based on matching of chromatographic retention times, MS1 values from detected features, and the resulting MS2 fragment ions to expected retention time ranges for the indicated lipid classes. Annotation was provided as MS1/MS2 when a cosine similarity score of >0.5 with at least two matched signals was observed. An m/z difference of 5 mDa or 3 ppm was allowed for precursor m/z matched, and fragments were allowed a 5 mDa or 5 ppm m/z difference. For MS1/rt matches, a 3 mDa or 3 ppm m/z tolerance was allowed, and the signal needed to elute within 0.3 min of the expected retention time for that lipid or lipid class. Observed peak heights were normalized to the sum of total annotated feature intensities per sample.

Super-resolution microscopy. Super-resolution images of EVs were acquired using the Nanoimager system (ONi) equipped with a 100×, 1.4 numerical aperture oil immersion objective. Purified C. albicans EVs were captured using desthiobiotynylated rabbit anti-EV polyclonal antibodies and stained using the lipophilic dye PanEV (ONi EV Profiler Kit 2). Imaging was done using the AutoEV function and image analysis was completed in the CODI online analysis platform (https://alto.codi.bio/).

Scanning electron microscopy. Catheters were segmented to expose the intraluminal surface. The biofilms were then fixed with 4% formaldehyde and 1% glutaraldehyde at 4°C overnight. Segments were then washed with PBS and treated with 1% osmium tetroxide for 30 min at 22°C. Samples were subsequently washed with a series of increasing ethanol dilutions (30 to 100%), followed by critical point drying and coating with platinum. SEM of samples was performed using a LEO 1530 microscope.

Statistics. Data sets of were analyzed using the nonparametric Kruskal-Wallis one-way analysis of variance, followed by uncorrected Dunn's multiple comparisons. Data processing was performed using GraphPad Prism 10 for Windows (64-bit, version 10.5.0 (774). $P<0.05$ considered significant.

Sex as a biologic variable. Only male rats were utilized for the study as growth on a biofilm device has not been shown to be impacted by sex or host immune response.

Study approval. Animal procedures were approved by the IACUC of the University of Wisconsin (protocol DA0031).

Data availability. The mass spectrometry proteomics data have been deposited to the ProteomeXchange Consortium via the PRIDE partner repository with the dataset dataset

accession: PXD076431. Supporting data values are presented in the file named CJI data for figures excel file.

Acknowledgements. These studies were supported by a grant from NIH/NIAID R01 AI073289-16 to DRA. We want to thank Ed Driggers and Duncan Holbrook-Smith (General Metabolics LLC) for help with lipidomics analysis.

References

1 Zarnowski, R. *et al.* Candida albicans biofilm-induced vesicles confer drug resistance through matrix biogenesis. *PLoS Biol* **16**, e2006872, doi:10.1371/journal.pbio.2006872 (2018).

2 Andes, D. *et al.* Development and characterization of an in vivo central venous catheter *Candida albicans* biofilm model. *Infect Immun* **72**, 6023-6031 (2004).

3 Nesvizhskii, A. I., Keller, A., Kolker, E. & Aebersold, R. A statistical model for identifying proteins by tandem mass spectrometry. *Anal Chem* **75**, 4646-4658 (2003).
