# Supplementary figures and images for: In vivo characterization of candida extracellular vesicles reveals unique infection pathway proteins
Source: JCI Insight. 2026 Apr 23;11(11):e198953. doi: 10.1172/jci.insight.198953 (PMC13317910; doi:10.1172/jci.insight.198953)

Fig 1 B

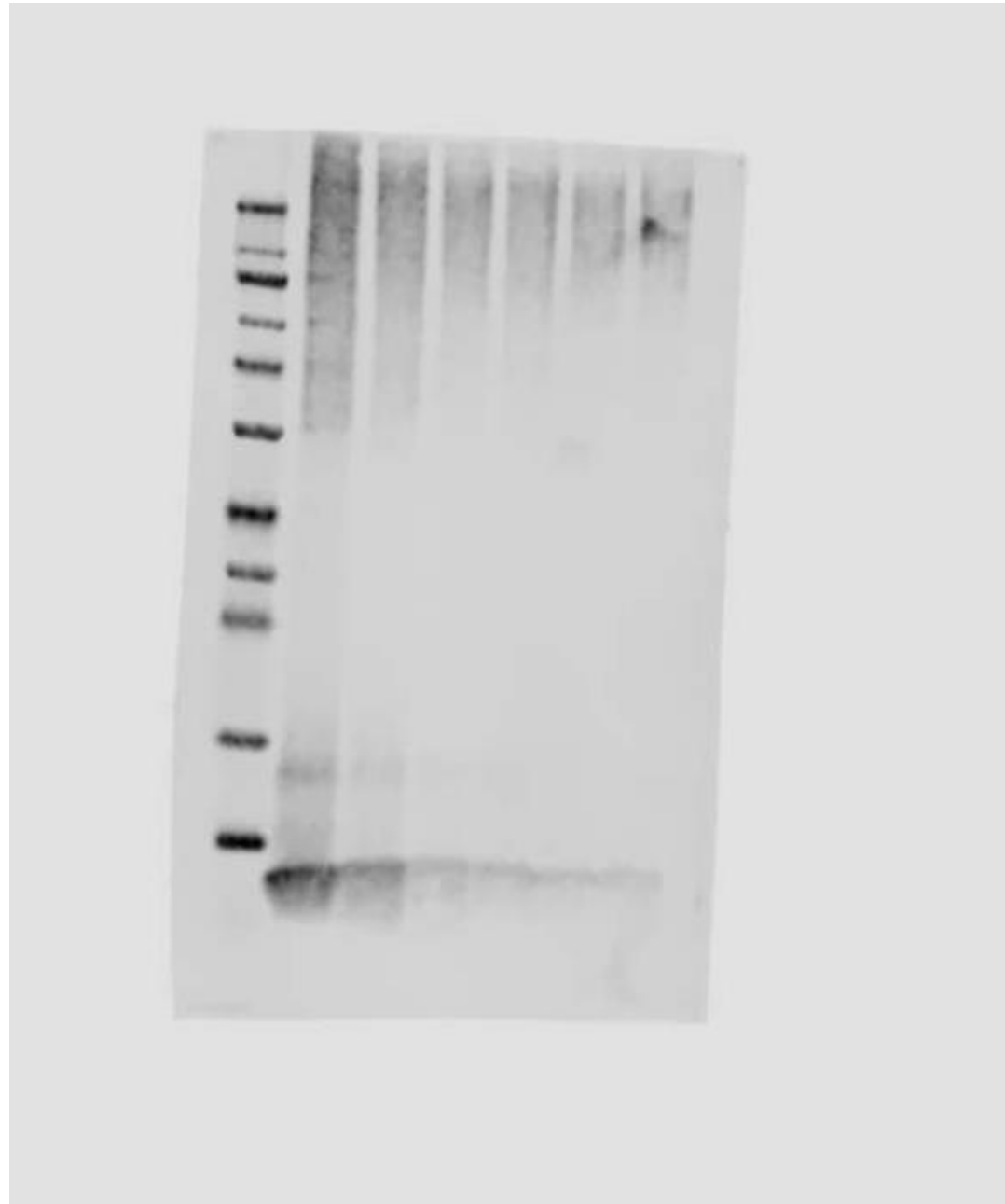

Fig 1D

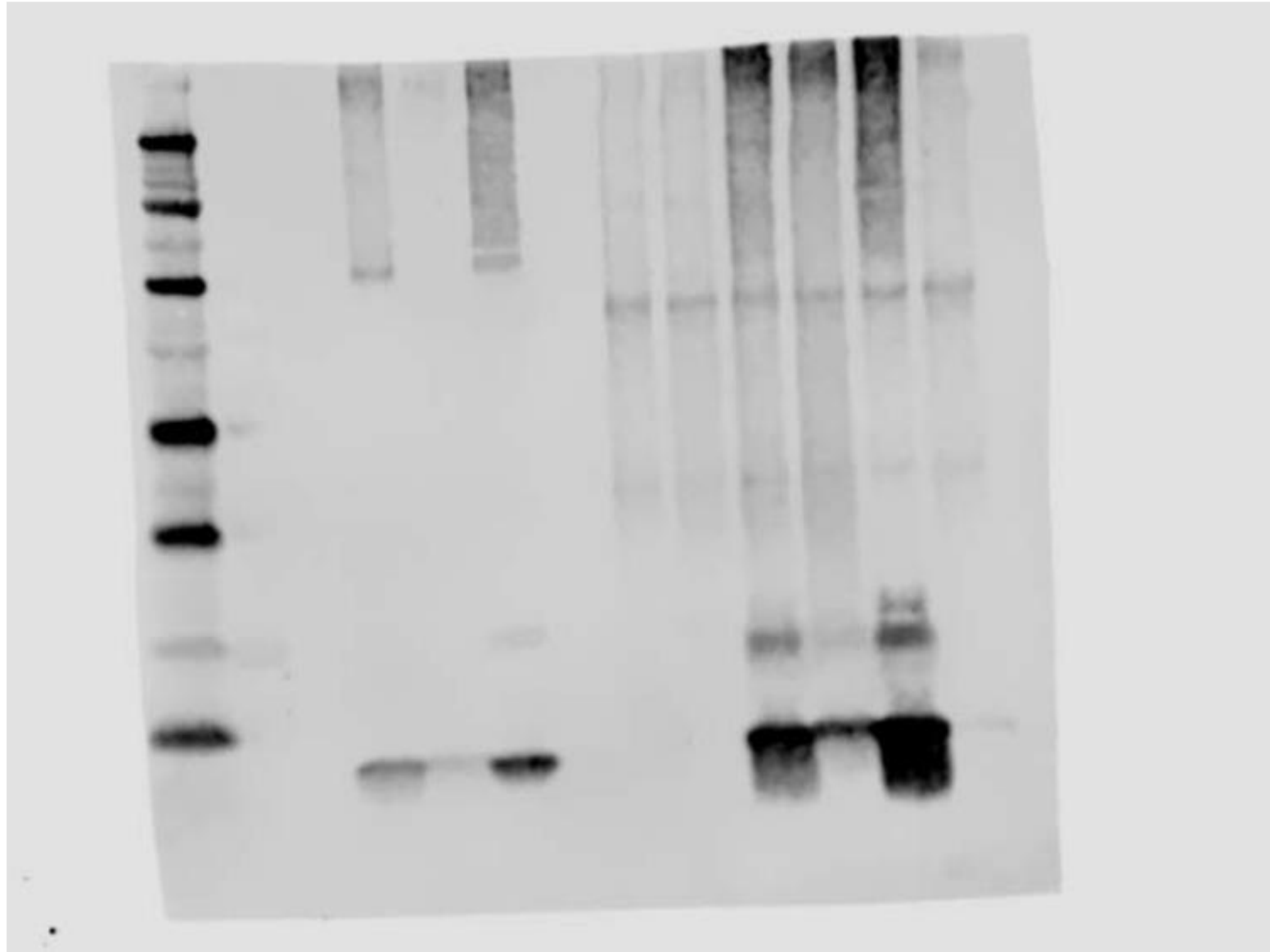

Fig S1A

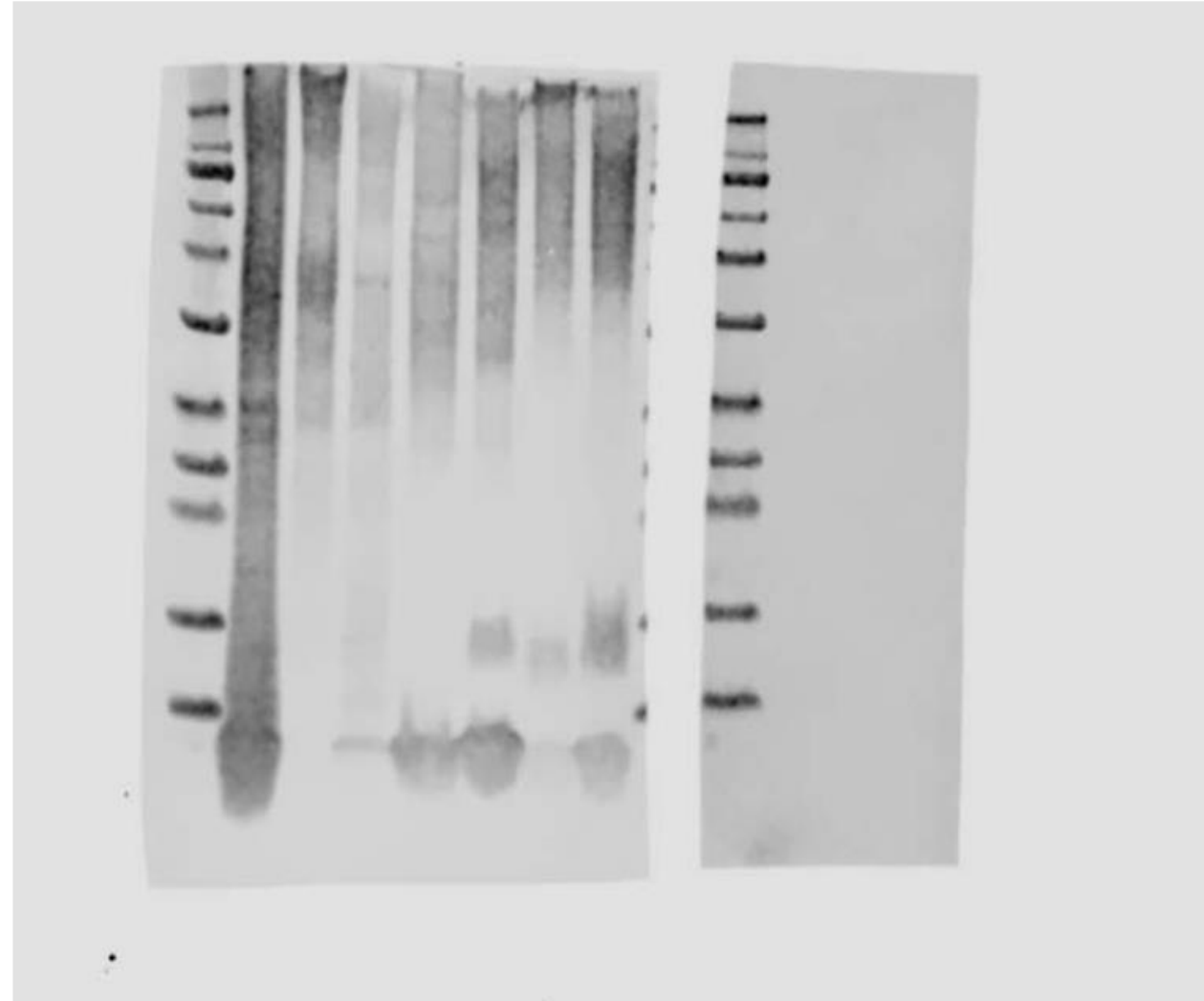

Fig S1 B

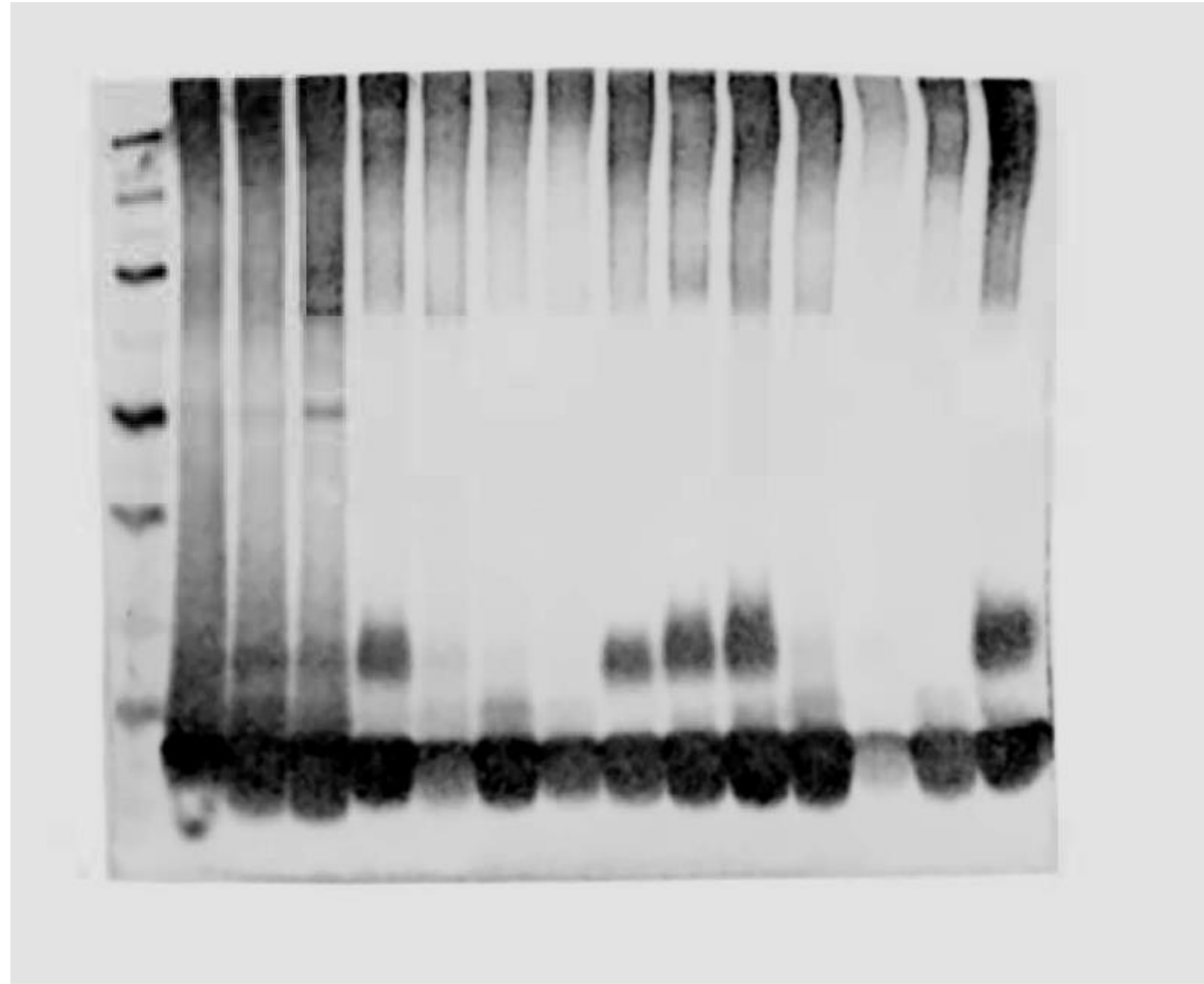

Supplement: Unedited blot and gel images [file jciinsight-11-198953-s003.pdf]
